# Production of L-carnitine by secondary metabolism of bacteria

**DOI:** 10.1186/1475-2859-6-31

**Published:** 2007-10-02

**Authors:** Vicente Bernal, Ángel Sevilla, Manuel Cánovas, José L Iborra

**Affiliations:** 1 Department of Biochemistry and Molecular Biology B and Immunology, Campus of Espinardo, University of Murcia, E-30100, Spain

## Abstract

The increasing commercial demand for L-carnitine has led to a multiplication of efforts to improve its production with bacteria. The use of different cell environments, such as growing, resting, permeabilized, dried, osmotically stressed, freely suspended and immobilized cells, to maintain enzymes sufficiently active for L-carnitine production is discussed in the text. The different cell states of enterobacteria, such as *Escherichia coli *and *Proteus sp*., which can be used to produce L-carnitine from crotonobetaine or D-carnitine as substrate, are analyzed. Moreover, the combined application of both bioprocess and metabolic engineering has allowed a deeper understanding of the main factors controlling the production process, such as energy depletion and the alteration of the acetyl-CoA/CoA ratio which are coupled to the end of the biotransformation. Furthermore, the profiles of key central metabolic activities such as the TCA cycle, the glyoxylate shunt and the acetate metabolism are seen to be closely interrelated and affect the biotransformation efficiency. Although genetically modified strains have been obtained, new strain improvement strategies are still needed, especially in *Escherichia coli *as a model organism for molecular biology studies. This review aims to summarize and update the state of the art in L-carnitine production using *E. coli *and *Proteus sp*, emphasizing the importance of proper reactor design and operation strategies, together with metabolic engineering aspects and the need for feed-back between *wet *and *in silico *work to optimize this biotransformation.

## Introduction

In general, in a biotransformation involving whole cells (either using growing, resting or even permeabilized cells), proper process operation and optimum production depends on the optimization of different variables. Among these, we may include (i) the physicochemical conditions of operation for maximum active enzyme concentration per unit of cell (protein synthesis, activity and/or stability), such as pH, temperature, osmolarity and the presence of activators/inhibitors, (ii) the concentration of both the catalyst and the substrate being used, (iii) the biocatalytic environment (i.e. reactor type), (iv) the optimization scheme, which, in turn, depends on the bioprocess studied, and (v) strain improvement strategies. Last but not least, enzymes involved in the bioprocess can be either constitutive or induced in certain conditions of cell growth. Optimization must look at both bioreactor and strain optimization and, in both fields, the development of suitable models would enable the correct decisions to be undertaken. In either case, process optimization requires a step by step scheme of variable optimization, as will be presented below.

L-carnitine (R(-)-3-hydroxy-4-trimethylaminobutyrate) is an ubiquitous compound, found in animal and vegetal tissues, as well as in microorganisms. The best characterized role of L-carnitine is the transport of long-chain fatty acids through the inner mitochondrial membrane: its involvement in the metabolism of heart, liver, muscle, brain and adipose tissues, as well as a certain role in sperm maturation, the immune system and connecting tissue have been well established [1]. Moreover, several clinical applications have been identified for this compound, which has led to increased demand worldwide and to the development of new production methods [1-3].

In the last 30 years, the feasibility of producing L-carnitine from waste products of the chemical industry, such as crotonobetaine or D-carnitine, by whole cell catalyzed biotransformations using *E. coli *and *Proteus sp *strains has focused the interest of both industry and applied science. The production of L-carnitine by biotransformation with *E. coli *and *Proteus sp. *strains is a good case-study to illustrate the feed-back between fundamental and applied research. The main aim of this review is to update the knowledge on bacterial carnitine metabolism (fundamental research) and the potential industrial application of its production methods (applied research), focusing on both bioprocess development and strain optimisation strategies.

### 1. Bacterial metabolism of L-carnitine

#### 1.1. Assimilation and/or biotransformation of L-carnitine by microorganisms

Although the role of L-carnitine is well established in eukaryotes, it is not so clear in bacteria [4]. The existence of uptake systems with different degrees of specificity in both gram positive and negative bacteria has been related with its protective properties. The ProP and ProU systems of *E. coli*, and the OpuC of *B. subtillis *have been shown to be involved in carnitine uptake under osmotic stress conditions [4-7]. Moreover, in some species, such as *Listeria monocitogenes*, the existence of betaine uptake systems has been related to protection against other stress conditions such as low temperatures [8] and to its ability to grow and survive in foods and to provoke infections *in vivo *[9].

**Figure 1:**
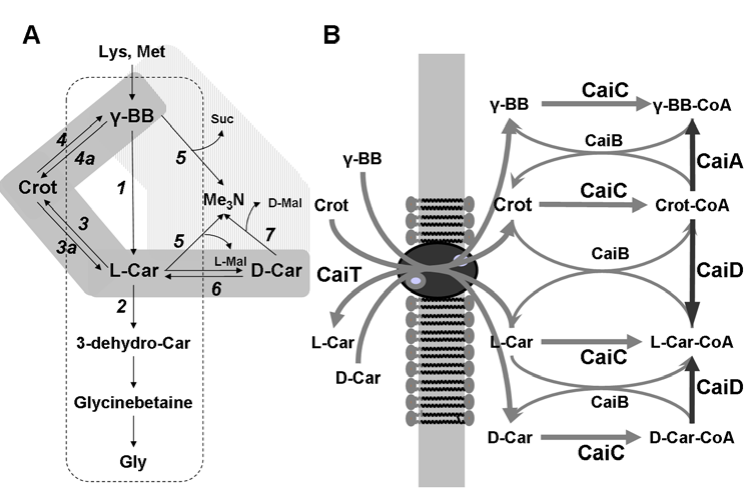
L-carnitine metabolism in bacteria. A) Schematic representation of the main pathways for L(-)-carnitine metabolization in bacteria. Shaded and striped reactions correspond to the pathways observed in Enterobacteria and *Acinetobacter *strains, respectively. Reactions in the dashed box correspond to *Pseudomonas *strains. Enzymes or "systems" involved: 1, γ-butyrobetaine hydroxylase; 2, L-carnitine dehydrogenase; 3, L-carnitine dehydratase; 3a, "carnitinyl-CoA hydrolase"; 4, crotonobetaine reductase; 4a, "γ-butyrobetaine dehydrogenase"; 5, monooxygenase; 6, "carnitine racemase"; 7, D-carnitine dehydrogenase. Adapted from [4]. B) Complete anaerobic biotransformation pathway for trimethylammonium compounds in *E. coli*. Adapted from [30]. Abbreviations: Crot, crotonobetaine; γ-BB, γ-butyrobetaine, L-Car, L-carnitine; D-Car, D-carnitine; Gly, glycine; 3-dehydro-Car, 3-dehydrocarnitine; Me_3_N, trimethylamine; Mal, malate; Suc, succinate; CaiT, L-carnitine/γ-butyrobetaine/crotonobetaine protein transporter; CaiA, CaiB, crotonobetaine reduction reaction; CaiB, CoA transferase; CaiC, L-carnitine/γ-butyrobetaine/crotonobetaine CoA ligase; CaiD, enoyl-CoA hydratase or carnitine racemase activity.

In addition to the protective roles exhibited after accumulating betaines, some bacterial species are also able to metabolize these trimethylammonium compounds under different conditions [4]. Depending on the species and the cultivation conditions (carbon and nitrogen sources, aerobic or anaerobic conditions), different pathways are involved in L-carnitine catabolism. The initial enzymes of the various catabolic pathways are induced by L-carnitine, but also partly by other trimethylammonium compounds. Different genera are able to degrade L-carnitine under aerobic conditions, assimilating both carbon and nitrogen in the molecule backbone. Some *Pseudomonas *species (like *Pseudomonas aeruginosa *A7244 and *Pseudomonas *sp. AK1) are able to grow aerobically on L-carnitine as the sole source of carbon and nitrogen (Fig. 1A). In these species, L-carnitine degradation starts by oxidation of the hydroxyl group with the concomitant formation of 3-dehydrocarnitine by an L-carnitine dehydrogenase (EC 1.1.1.108) [10]. *Pseudomonas *sp. AK1 is also able to grow on γ-butyrobetaine, which is an intermediate in the degradation pathway [11]. This pathway has similarities with the biosynthetic pathway of L-carnitine in eukaryotes (Fig. 1A).

Furthermore, some species like *Acinetobacter calcoaceticus *69/V are not able to assimilate nitrogen from the L-carnitine skeleton and degradation occurs with the stoichiometrical formation of trimethylamine [12] (Fig. 1A). This bacteria is able to metabolize L-carnitine, L-O-acylcarnitines and γ-butyrobetaine as sole carbon source. D-carnitine can also be metabolized but only in the presence of L-carnitine to act as an inducer [12]. The stereoselectivity observed in the metabolism of this strain could be a result of the existence of two separate transport systems for D- and L-isomers, since the wild-type strain *A. calcoaceticus *ATCC 39647 exhibits enantiomer discrimination due to the differential cell membrane permeability [13].

Finally, Enterobacteriaceae, such as *E. coli*, *Salmonella typhimurium*, *Proteus vulgaris *and *Proteus mirabilis*, do not assimilate the carbon and nitrogen skeleton of trimethylammonium compounds, but are able to metabolize carnitine, *via *crotonobetaine, to γ-butyrobetaine [4]. The presence of adequate carbon and nitrogen sources during anaerobic (and in some cases also aerobic) growth is necessary for proper expression of the genes for components of the biotransformation machinery. Further, the biotransformation can also occur in the absence of nutrients, as has been shown in studies with resting cells [14].

Although the D-enantiomer does not exist in nature, interestingly, various bacteria are able to further catabolize or biotransform it [4,14].

#### 1.2. L-carnitine pathway in Enterobacteria: *E. coli *and *Proteus sp*

Initially, a two-step pathway was proposed for L-carnitine metabolization in *E. coli *and *Proteus *sp. strains, including two enzyme activities: L-carnitine dehydratase (CDH, EC 4.2.1.89) and crotonobetaine reductase (CR, EC 1.3.99) [15,16] (Fig 1A). Subsequently, carnitine racemase activity (CRac), interconverting the D- and L-isomers, was also described [17]. The cloning of the *cai *operon in *E. coli *[18] and *Proteus sp. *[19] showed a higher degree of complexity, and also pointed to similar organization and high degree of homology between both strains. Six ORFs were found and functions were first assigned on the basis of sequence homology, and later experimentally demonstrated (Table 1). Recent studies have deciphered the roles of almost all *cai *operon gene products (Fig 1B). Thus, CaiT is a highly specific transporter working as antiporter, allowing substrate and product exchange [20,21]. The biotransformation occurs at the level of CoA derivatives [22,23], while the initially described CDH and CR activities were shown to depend on two proteins (enoyl-CoA hydratase, CaiD, EC 4.2.1.89, and crotonobetainyl-CoA reductase, CaiA, EC 1.3.99, respectively) both needing the joint action of a transferase (crotonobetainyl-CoA:carnitine CoA transferase, CaiB, EC 2.8.3) to allow the CoA-cycling between products and substrates of the biotransformation [24,25]. CaiC (betainyl-CoA ligase, EC 6.2.1) has been shown to catalyze the synthesis of the CoA-derivatives of trimethylammonium compounds, which are the substrates of both CaiD and CaiA activities [26]. The only function which remains unconfirmed is that of CaiE, although early overproduction experiments pointed to an activation of the CaiD/CaiB function (CDH activity, Fig. 1A), suggesting a possible role as a cofactor of these enzymes [18].

**Table 1:** Comparison of *cai *operon codified proteins in *E. coli *and *Proteus sp. *strains [4,15,19]

Gene	Protein length (aa)		Function of gene product	Homology (%)	Reference
				
	*E. coli*	*Proteus sp.*			
*caiT*	504	504	Transport protein*	88	[20,21]
*caiA*	380	380	Crotonobetainyl-CoA reductase	93	[16,65]
*caiB*	405	406	Betainyl-CoA transferase	86	[22,24,25]
*caiC*	524	518	Betainyl-CoA ligase*	69	[26]
*caiD*	297	261	Crotonobetainyl-CoA hydratase	82	[24,25,60]
*caiE*	203	197	Unknown	76	[15,19]
*caiF*	131	130	Transcriptional regulator*	51	[19,27]

(*) In *Proteus sp. *strain, function postulated on the basis of sequence similarities [19].

A seventh ORF, the *caiF *gene, which is located downstream of the *cai *operon and is transcribed in the opposite direction to this, has been cloned [19,27] (Table 1). The product is a transcriptional factor which, in *E. coli*, is synthesized when cells are grown under anaerobiosis and in the absence of glucose as carbon source. In the presence of L-carnitine, CaiF becomes active, and is able to induce the expression of *cai *genes [28]. High regulation of this metabolism has been demonstrated in *E. coli *and *Proteus sp. *strains. Transcription of the *cai *operon is induced during anaerobic growth in the presence of L-carnitine and occurs as a polycistronic mRNA. The expression of *caiF-lacZ *and *caiT-lacZ *fusions is enhanced 20- and 200-fold, respectively, when cells are grown under anaerobic conditions [27,29]. In terms of enzyme activities, when cells are grown under aerobiosis, carnitine dehydratase (CDH) has been reported to be 3-fold higher, while no crotonobetaine reductase (CR) can be detected [30]. As regards the expression mechanism, the activator of carbon catabolic operons, CRP, is required for induction. In addition, the histone-like H-NS protein and the σ^S ^factor (RpoS), which is involved in gene regulation in stationary-phase, exert a repressive effect on carnitine metabolism [18,29]. The mechanism of repression remains unknown, although there is evidence that it probably operates at the level of *caiF *expression [27].

It should be mentioned the fact that *caiF *is the gene having the lowest homology between both species (Table 1) and that alterations in its promoter region have been proposed as being responsible for the very different regulation of this pathway in *Proteus sp. *[19].

Another operon composed of four ORFs was found in *E. coli *at the 5' end of the *cai *locus in *E. coli*. It was shown to be co-transcribed from the same promoter/operator region [31] and the corresponding proteins displayed significant sequence homology with polypeptides encoded by the *fix*ABCX operon from *Azorhizobium caulinodans *and *Rhizobium meliloti*, and were therefore named *fix*. This operon has been confirmed as being involved in electron transfer to crotonobetaine [32]. Deletion studies have also shown that part of the *fix *sequence is necessary for the proper expression of *cai *operon [29].

Despite all this accumulated knowledge, the precise function of this reaction sequence in Enterobacteria remains unknown. Seim et al., postulated that crotonobetaine serves as an external electron acceptor of anaerobic respiration similar to nitrate, fumarate and trimethylamine-N-oxide [33,34]. The stimulation of anaerobic growth in Enterobacteria by crotonobetaine and the suppression of this reaction by nitrate or glucose certainly support this hypothesis. The functional characterization of the CaiT transport system as an antiporter for L-carnitine and γ-butyrobetaine [20] is also in accordance with this idea and would explain why this kind of transporter cannot be involved in osmoprotection [6,35]. However, the induction under aerobic conditions of carnitine metabolism in several Enterobacteriaceae [23,36], including *Proteus *strains, suggests a possible loss of function due to mutations affecting the regulation of this pathway. In addition to these functions, some results show that microorganisms of the gastrointestinal tract may play a role in lowering the concentration of dietary L-carnitine [4,33].

### 2. Overview of current methods for L-carnitine production

#### 2.1. Chemical methods for L-carnitine production

Much research effort has focused on the development of methods for the industrial scale production of L-carnitine. Numerous chemical procedures can be found in the literature involving asymmetric synthesis [37-39]; chemical multistep racemization [40]; resolution through diasteroisomeric derivatives [41-43]; microbiological or enzymatic techniques [44-47] and the use of chiral starting materials [48-50]. For instance, the method developed by Bellamy et al. [49], consisted of six steps which, using as starting material (R)- and (S)-malic acid, respectively, specifically obtaining both enantiomers. More recently, Marzi et al. [51] described an enantioselective synthesis using achiral glycerol as starting material and a chiral auxiliary. However, few of these chemical procedures are of practical use on an industrial scale because of the number of steps, and the need to use of chiral starting materials or chiral auxiliaries. The classical industrial method for the synthesis of carnitine generates a racemic mixture, with D-carnitine as waste product [41].

#### 2.2. Biotechnological methods for L-carnitine production

The potential advantages of biotechnological methods employing both enzymes and microorganisms have encouraged extensive research into the microbial metabolism of L-carnitine and its derivatives [2,4,46,47,52]. These methods mostly aim at using industrial waste products (such as D-carnitine, crotonobetaine or γ-butyrobetaine) as substrates. In addition, biotechnological procedures have other advantages over chemical processes. Compared with the classical chemical method of racemate synthesis involving resolution of the enantiomers, the enantioselective production of racemate by fed-batch cultivation of a *Rhizobium*-like strain, involves 50% less total organic waste, 25% less waste water and 90% less waste for incineration [2,53].

The most commonly used starting materials for the production of L-carnitine are achiral precursors (mostly crotonobetaine, γ-butyrobetaine and 3-dehydrocarnitine) or racemic mixtures (such as D, L-acyl-carnitine, D, L-carnitinamide and D, L-carnitine) [2,4,41,46,52]. A great variety of microorganisms can be used for these biotransformations (Table 2). Since the early 1980s, many companies worldwide have patented bioprocesses for L-carnitine production (Seitetsu, Kyowa Hakko, Chou Kaseihih, Toyo Jozo, Ajinomoto, Sigma Tau, Lonza, Nippon Pet Food, Yakult Honsha, Elf Aquitaine, Sanofi) [2]. As an example, while bioprocesses developed for commercial production of L-carnitine by Sigma Tau (Italy) are based on the biotransformation of crotonobetaine by *E. coli *and *Proteus mirabilis *strains, Lonza (Switzerland) uses γ-butyrobetaine as starting material and a derivative of the HK4 strain. This latter strain was isolated from a soil sample and its pathway from γ-butyrobetaine to L-carnitine is analogous but not identical to fatty acid degradation. Taxonomically, this bacteria would be between *Agrobacterium *and *Rhizobium*, close to *Rhizobium meliloti *[52]. This strain was able to grow on L-carnitine as the sole source of carbon and nitrogen under aerobic conditions, while the degradation of L-carnitine was blocked by frameshift mutagenesis, giving rise to a derivative strain, HK13, lacking L-carnitine dehydrogenase [53]. Similarly, several Cai proteins of *E. coli *show a high degree of homology with enzymes involved in fatty acid degradation, such as acyl-CoA dehydrogenase and CaiA, acetate-CoA ligase and CaiC, and enoyl-CoA hydratase and CaiD [18].

**Table 2:** Microorganisms used for L-carnitine production from chiral and achiral substrates and the enzyme activities involved. Adapted from [2].

	Substrates	Activities involved	Microorganism
**Achiral precursors**	Crotonobetaine	*L-carnitine dehydratase*	*E. coli, Proteus mirabilis, Acinetobacter lwoffi, Achromobacter xylosoxydans*
	γ-butyrobetaine	*γ-butyrobetaine hydroxylase*	HK4, HK13, HK1349 (taxonomically between *Rhizobium *and *Agrobacterium*), *Saccharomyces cerevisiae, Penicillium, Rhizopus, Mucor, Actinomucor, Neurospora, Aspergillus, Achromobacter, Pseudomonas, Nocardia crassa*
	3-dehydrocarnitine	*L-carnitine dehydrogenase (NADH dependent)*	*Agrobacterium, Pseudomonas*
**Racemic mixtures**	D, L-carnitinenitrile	*Nitrilase*	*Corynebacterium sp.*
	D, L-acyl-carnitine	*Acyl-L-carnitine esterases*	*Fusarium oxysporum sp. lini, Corynebacterium, Bacillus, Pseudomonas*
	D, L-carnitineamide	*Carnitine amidase*	*Pseudomonas sp., *DSM 6320 *(Agrobacterium or Sphingomonas sp.)*
	D, L-carnitine	*Carnitine racemase*	*Pseudomonas sp., E. coli*
		*D(+)-carnitine assimilation*	*Acinetobacter calcoaceticus Acinetobacter lwoffi*

Although the productivities reported for *E. coli *strains are not the highest found in the literature, the genomic and metabolic constraints controlling L-carnitine metabolism are well-characterized. In addition, L-carnitine production using this microorganism has the main advantage of its well demonstrated capacity for high density cultivation [54,55], its resistance to immobilization [56,57] and to environmental stresses during bioprocesses [30,35] and the availability of well established molecular biology techniques, allowing the application of top-down strategies for bioprocess optimization. Consequently, the main factors affecting the production of L-carnitine with both wild type and genetically engineered *E. coli *strains will be reviewed. In addition, comparisons will also be made with *Proteus sp. *which possesses a very similar carnitine metabolism [19,23], somewhat better bioprocess performance [58,59], and promises very interesting improvements.

### L-carnitine production with *E. coli *and *Proteus sp. *strains: bioprocess optimization

#### 1. Effect of inducers and oxygen on metabolizing activities

The capacity of *E. coli *and *Proteus sp. *cells to produce L-carnitine by biotransformation of either crotonobetaine or D-carnitine has been shown to be inducible. The proper biotransformation capacity is only induced in the presence of compounds such as crotonobetaine, D-carnitine or even L-carnitine in the growth medium [17]. Maximal biotransformation capacity (determined as specific L-carnitine dehydratase activity, CDH) is induced in the presence of 5 mM crotonobetaine in anaerobiosis. In addition, under production conditions, CDH activity remains high since both the substrate and the product of the bioprocess are inducers. Both *Proteus *and *E. coli *are able to synthesize the above mentioned L-carnitine metabolism-dependent enzymes: carnitine dehydratase and crotonobetaine reductase [16,25,60]. L-carnitine production can reach 50–65% (molar yield), depending on the strain, cells and substrate concentration and the running conditions. In addition, variable amounts of the side product γ-butyrobetaine can be detected (Fig. 1) [14,25,61,62]. In fact, γ-butyrobetaine is always produced, especially during the growth phase, but also, at a lower rate during the resting cell production process.

The effect of aerobic/anaerobic conditions on the induction of L-carnitine metabolism and on the biotransformation performance was also explored. For this, cells were induced in aerobic and anaerobic conditions and both cell cultures were used for biotransformation in resting. The biotransformation using resting cells was also performed under both aerobic and anaerobic conditions (100 mM crotonobetaine, 4 g l^-1 ^biomass, pH 7.5 and 37°C). When non genetically engineered *E. coli *strains were grown aerobically, no L(-)-carnitine production was observed. In the case of *E. coli *K38 pT7-5KE32 strain, which overexpresses both CaiF and CaiD proteins, when growth and induction were performed aerobically, up to 1.5 g L^-1 ^h^-1 ^L-carnitine (yield: 38.2%) were produced, in contrast to the 11.3 g L^-1 ^h^-1 ^(yield, 70.1%) produced in anaerobic conditions [61]. Moreover, the best results were obtained using anaerobiosis for the carnitine metabolism induction phase and aerobiosis for the biotransformation of crotonobetaine into L-carnitine [14]. In the case of the non-transformed *E. coli *O44K74 strain, when comparing anaerobic and aerobic conditions for the biotransformation using cells grown under anaerobic conditions, productivities of 0.18 g L^-1 ^h^-1^, and 0.21 g L^-1 ^h^-1 ^(34 and 39% molar yield) respectively, were obtained [14]. Up to a 53.2% L-carnitine yield was obtained with *E. coli *pT7-5KE32 after 2 h incubation in anaerobic conditions, which meant a productivity of 10.3 g L^-1 ^h^-1^, whereas aerobic biotransformation provided 70.1% product yield in 1 h, the productivity being 11.3 g L^-1 ^h^-1 ^[61]. Thus, both yield and productivity were higher when the cells were induced during anaerobic growth, meaning that anaerobiosis was the preferred condition for carnitine metabolism induction, while aerobic conditions yield increased biotransformation performance.

A more in-depth analysis of the effect of oxygen levels permitted conditions to be optimized [63,64] and showed that γ-butyrobetaine production was dramatically decreased when the biotransformation was performed under aerobic conditions as a consequence of the inhibition of crotonobetaine reductase by oxygen [14,16,63,65]. Other compounds known to be electron acceptors for *E. coli *were checked for their ability to inhibit γ-butyrobetaine production and enhance L-carnitine yield under anaerobic conditions, but worthwhile results were only obtained with fumarate [14].

In the case of the *Proteus sp*. strain, γ-butyrobetaine production has been shown to be much lower both with growing and resting cells, although has been observed in high cell density reactors, in which oxygen limitations can occur [58]. Crotonobetaine reductase activity has been detected in cell free extracts, meaning that impaired exportation of γ-butyrobetaine might occur [19,23].

#### 2. Biotransformation with D-carnitine or crotonobetaine as substrates

As already mentioned, both D-carnitine and crotonobetaine can be used as biotransformation substrates. Nevertheless, the different levels of carnitine dehydratase and carnitine racemase activities explain why the highest productivities are achieved with crotonobetaine [63]. In addition, the production of L-carnitine from D-carnitine is only possible if production experiments are performed under fully or partially aerobic conditions with both growing and resting cells [14,63]. Increased productivity was obtained using a genetically modified strain overproducing the multifunctional protein CaiD, which is part of both carnitine dehydratase (CDH) and carnitine racemase (CRac) activities [61]. In this overproducing strain, *E. coli *K38 pT7-5KE32, the level of the respective enzyme activities responsible for the biotransformation correlated well with the productivity obtained using each substrate, since carnitine dehydratase activity approximately doubled the level of carnitine racemase.

#### 3. The implications of transport phenomena for bioprocess optimization: three different strategies

Many works have underlined the importance of the kinetics of substrate uptake and product efflux for the development of bioprocesses using whole cells [66]. The engineering of transport phenomena allows for the improvement of bioprocesses through various strategies.

Although membrane integrity is necessary for maintaining the chemical independence of the cell from the extracellular medium, and is essential for cell survival, many so-called cell permeabilization methods have been developed to improve the biotransformation process [67,68]. These methods can greatly affect membrane structure and low (or even high) molecular weight compounds may leak out of the cell (e.g. cofactors), not only compromising cell viability, but also bioprocess performance. However, in bioprocesses in which growth and biotransformation are uncoupled, cell proliferation is not necessary to maintain productivity.

**Figure 2:**
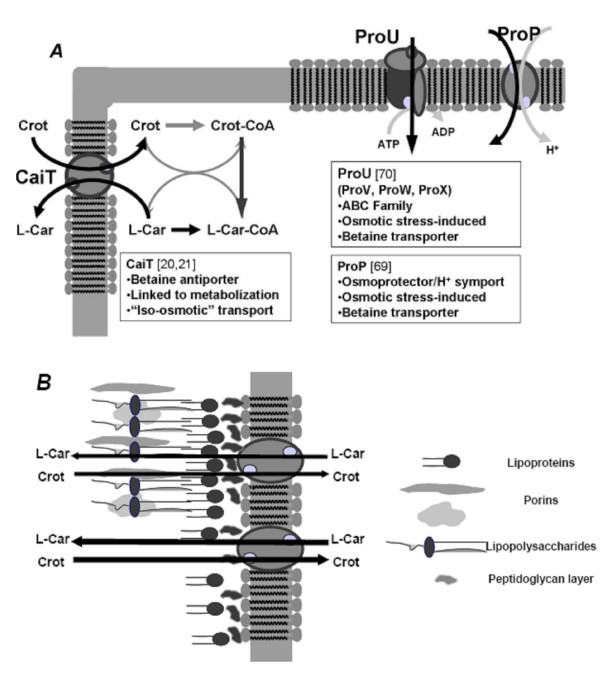
L-carnitine transport systems in *E. coli *strains. A) Main characteristics of L-carnitine transporters: CaiT: carnitine/crotonobetaine/γ-butyrobetaine antiporter [20]; ProU and ProP, osmotic stress related transporters [6,69]. B) Effect of permeabilizers on cell envelope and outer membrane Adapted from [76].

Besides, the molecular composition of bacterial membrane can be engineered in order to increase the occurrence of membrane transporters. L-carnitine, as well as other betaines, are osmoprotectants, which means that bacteria can accumulate them intracellularly in order to allow them to cope with high extracellular amounts of osmotically active compounds [6,35,69,70]. Thus, cells were subjected to salt stress conditions in order to induce the expression of the betaine transporters involved in osmoprotection, with the aim of tuning up cellular L-carnitine uptake rates [6,71] (Fig. 2A). Finally, the application of molecular biology techniques allows specific targeting of the overexpression of selected genes, such as that of the antiporter CaiT [72].

##### 3.1. Permeabilization of *E. coli *and *Proteus sp. *cells

Gram-negative bacteria like *E. coli *possess a mechanically strong cell envelope made up largely of peptidoglycan, which gives the cells shape and protects them from osmotic lysis (Fig 2B). Outside this layer, there is an additional outer membrane (OM), whose role is mainly protective and which is less selective and more permeable than the cytoplasmic membrane. The OM functions as an efficient barrier against hydrophilic macromolecules and hydrophobic substances due to a specific lipopolysaccharide layer on the membrane surface. The integrity of the cell envelop (in general) and even of the OM (gram-negative bacteria) can be disturbed by permeabilizers, such as detergents (Triton X-100 and Tween), EDTA, organic solvents (toluene, lactic acid and alcohols) and certain polycationic substances (such as polyethylenimine, polymyxin and its derivatives, polylysines and protamine) [73-76].

**Figure 3:**
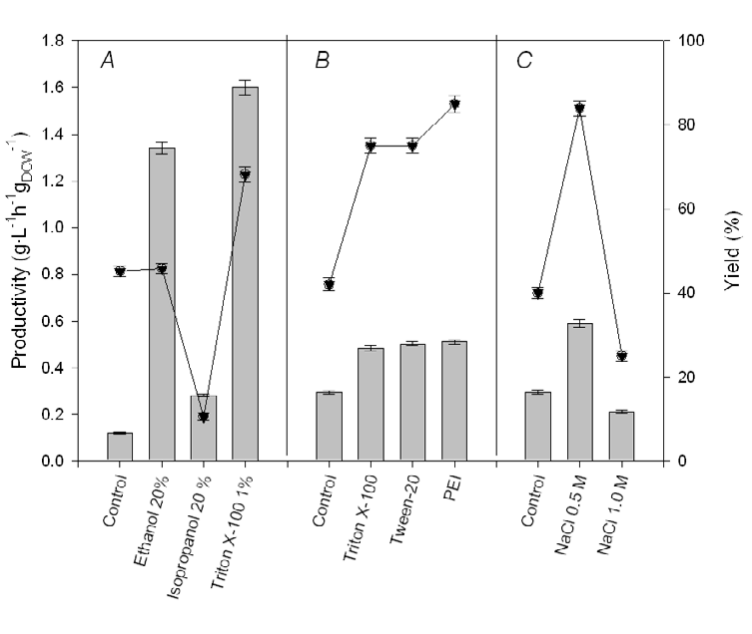
Effect of different transport engineering strategies on L-carnitine production. Permeabilization of *Proteus sp*. (A) and *E. coli *O44K74 (B) cells using detergents and organics. L-carnitine production by *E. coli *O44K74 under salt stress conditions (C). Bar represents productivity and lines the yield. PEI stands for polyethylenimine. Adapted from [76,79].

As previously seen in many other biotechnologically relevant systems, the substrate (crotonobetaine) uptake and product (L-carnitine) efflux are controlled by diffusion through both the outer membrane (OM) and cell envelope, together with the membrane transport system itself (CaiT) (Fig 2B) [77]. Cell damage and integrity during permeabilization has been assessed by fluorescent probe uptake assays (such as 1-phenylnaphthylamine or NPN) [78], protein cell leakage, transmission electron microscopy (cell structural alterations) and transport assays with L [*N-methyl*^14^C]carnitine (specific carrier transport studies) [76]. Both *E. coli *and *Proteus sp. *cells were treated for different times and at different permeabilizer concentrations, during batch cell growth. After permeabilization treatment, the culture broth was centrifuged and cells were resuspended in the biotransformation medium (crotonobetaine in phosphate buffer) for 48 h. For *Proteus sp.*, Triton X-100 resulted in higher conversion and productivity values than those of the control (Fig. 3A). An increase in L-carnitine molar yield of more than 50% was achieved in both growing and resting biotransformation media [79]. For *E. coli *O44K74, an almost 100% increase in yield was observed (Fig. 3B) [76], with polyethylenimine (PEI) being the best permeabilizer. Transmission electron microscopy of *Proteus sp. *and *E. coli *cells after permeabilization demonstrated that the cell wall was affected while the membrane remained intact, meaning that crotonobetaine/L-carnitine transporter could work faster and far more efficiently (Fig. 2B).

More recently, a new method for the permeabilization of *E. coli *has been developed. The mutation of *lpp*, which encodes Braun's lipoprotein, a major component of the outer membrane, does not affect cell growth and metabolism and increases the rate of whole cell-catalyzed reactions in which substrate diffusion is known to be a controlling factor. The authors reported a 30% increase in the molar yield of L-carnitine production (up to 56%) when using resting cells carrying this mutation [77].

It should be remarked that by permeabilizing the cell, a biotransformation unit containing a cofactor-regenerating system (CaiC and CaiB enzymes, Fig. 1B) would be deviced, the efficiency of the biocatalytic process being increased. Thus permeabilized cells offer an interesting alternative to the often-addressed problem of coenzyme regeneration.

##### 3.2. Overproduction of CaiT

In the case of trimethylammonium compounds metabolism, the expression of *cai *operon leads to the presence in the membrane of the specific antiporter CaiT. In *E. coli*, CaiT protein has been stated to be highly specific and able to work independently of cellular energy [20]. However, the cell state may affect the degree of multimerization of the transporter, which has been proposed as being involved in its activity [21]. The CaiT protein of *Proteus sp. *is unable to excrete γ-butyrobetaine, thus explaining why this compound is not detected extracellularly in this production strain (Bernal et al., unpublished results). The increase in carnitine production by permeabilized cells [76,77,79] or after salt treatment [71], underlines the control that transport exerts on this biotechnological process.

When *caiT *was cloned and overexpressed in *E. coli *LMG194 (a standard laboratory strain), an almost 3-fold increase in L-carnitine molar yield was obtained with both growing and resting cells [72]. However, the level of production of L-carnitine, in the engineered strain was still far from that of the *E. coli *O44K74 strain (6.9 vs 37.5 mmol·g_DCW_^-1^). Thus, although transport limits L-carnitine production, it is not the main factor controlling the biotransformation in laboratory strains [26]. However, further work is necessary to determine the effect of the overexpression of other transporters on L-carnitine production. Furthermore, the occurrence of metabolite transporters in bacterial genomes has to be considered in the possible light of a physiological role. In the case of amino acid production strains, uptake may be physiologically significant, while the interest of excretion is less obvious, only being explained in certain cases by overflow metabolism, limited catabolism and deregulated anabolism, a fact that has been considered in strain development studies [66]. In this case, CaiT is the only transporter involved in L-carnitine uptake under standard salt concentrations (unpublished data), while it has already been demonstrated not to be involved in osmoprotection [6], which is a consequence of the antiport mechanism of transport [20].

##### 3.3. Effect of salt stress on the biotransformation

Despite the importance of osmotic stress phenomena in the determination of cell survival and growth in important systems of study such as food, water and bioprocesses, there is little information available on their effect on cell metabolism [80]. Betaine uptake systems have been described in *E. coli*, which are involved in osmoprotection. In fact, the intracellular accumulation of osmotically active compounds (compatible solutes) allows the cell to cope with several stress conditions [7]. In *E. coli*, the ProU and ProP (Fig. 2A) systems have been described as being regulated to deal with these stress conditions, both being energy-consuming [7,69,70]. Despite this, CaiT is not involved in stress adaptation [6] and in stress situations carnitine metabolization is inhibited in growing cells [4,71]. However, metabolism alterations that follow osmotic stress adaptation improve L-carnitine production in resting cells, and perturbation studies revealed a regulation in the main pathways involved in ATP production and in the balancing of anabolism and catabolism [35]. Furthermore, the effect of salt stress on biotransformations should be considered, since both substrates and products are osmoprotectants. Therefore, the biotransformation of D-carnitine or crotonobetaine into L-carnitine by resting cells of *E. coli *strains under different salt stress conditions was studied.

Cells were grown and the biotransformation assays carried out with resting cells in aerobiosis in the presence of different NaCl concentrations. The maximum L-carnitine yield obtained for control resting cells was 40–45%, depending on the substrate and its concentration [71]. L-carnitine production from crotonobetaine was enhanced when the NaCl concentration was increased to 0.5 M, reaching 85–90% yield for *E. coli *pT7-5KE32 (Fig. 3C); no improvement was obtained at higher NaCl concentrations. On the contrary, the effect of salt stress on the biotransformation of D-carnitine in L-carnitine was deleterious (yield control values were 20–22% for both strains), since the carnitine racemase activity is negatively affected by high ionic strength when assayed in cell-free extracts [4].

#### 4. Biocatalyst optimization: reuse and reactivation of freely suspended and immobilized cells

When using non-growing bacterial cells as biocatalyst, the biomass growth phase is usually more time consuming than the biotransformation phase. The possibility of reusing cells allows to greatly increase bioprocess productivity, while diminishing the amount of growth medium needed for catalyst production. In addition, in the successive cycles of operation, the biotransformation capacity of cells may be lost, as seen with *Agrobacterium sp*., *Proteus sp. *and *E. coli *cells [59,62,81], which suggests cofactor leak or degeneration or even a decrease in the cell enzyme level. Thus biocatalyst regeneration should be regarded as essential.

In the case of *E. coli *cells, since the biotransformation occurred in a short period of time (1–10 h, depending on cell strains), while the growth phase was slow (15 h), the possibility of reusing the resting cells was checked. With the more slowly biotransforming strains (non transformed *E. coli *O44K74, for instance) a decrease in L-carnitine productivity during successive cycles of biotransformation has been observed. However, the biotransformation capacity could be recovered if after each biotransformation cycle (16 h) a regeneration cycle was followed (8 h incubation in fresh growth medium) [62]. The analysis of resting cells state by means of flow cytometry revealed a decrease in cell viability during the first 15–24 h, coinciding with the end of the biotransformation [82]. Thus, de-energization of resting cells and, probably, also cofactor leak or degradation, are a major drawback for continuous use in biotransformation.

A different scenario was found in the case of the genetically engineered strain *E. coli *pT7-5KE32. This engineered strain overproduce the L-carnitine dehydratase activity [61], thus allowing for a very short run time (1 h). During successive cycles of biotransformation, product yield was maintained over 50% for eight reuse steps, only decreasing 16% during this time [79]. This reuse of biomass permitted about an 8-fold increase in the amount of L-carnitine produced per gram of cells. Thus, the short run time also allowed the reuse of cells, since cell viability was not affected, and the productivity remained well above 50% after eight reuse cycles. A single biotransformation process took 16 h, (14 h for cell growth and 2 h for both cell harvesting and the biotransformation process), to produce 2.7 g L-carnitine per gram of biomass. After eight reuse cycles, 20 g L-carnitine g^-1 ^cells were produced in 30 h; this means that seven growth phases were avoided, total process time being reduced from 128 to 30 h. The amount of L-carnitine produced per gram of cells was increased 8-fold, consequently improving overall efficiency of the bioprocess [79].

Moreover, with *Proteus mirabilis *a decrease in productivity was also observed during successive cycles, although this has been demonstrated to be partially attenuated when cells are immobilized, meaning that mechanical stress might also be involved [59]. In addition, cell immobilization is an advantage for cell reuse [56,59].

Therefore, the use of recombinant microorganisms together with the reuse of the biocatalyst (immobilized or not) always leads to increased efficiency of these production systems.

#### 5. Biotransformation using growing and resting cycles in immobilized cell reactors for cell activation

In order to increase the productivity of the biotransformation, immobilization methods can be applied. The adequate selection of solid support material is necessary to attain high productivities. When using packed bed reactors with solid supports, such as polyurethane foam and glass beads, higher L-carnitine productivities have been reported (1.8 g·l^-1^·h^-1^, 26% yield) [56] than when using polyacrylamide (0.3 g·l^-1^·h^-1^) [83]. In the case of *Proteus mirabilis *in poly (vinyl alcohol) cryogels, yields of over 40% and a productivity of 0.72 g L^-1 ^g^-1^_biomass _h^-1 ^have been reported (Table 3) [59]. Moreover, these processes were run by alternating growing and resting cycles so that cell activation would allow continuous production processes (see previous section).

**Table 3:** L-carnitine productivities reported in the literature for different batch and continuous systems with growing and resting cells. See text for further details.

**Batch Growing Cells**				
**Strain**	**Productivity**	**Molar yield (%)**	**Comment**	**Ref.**

*E. coli *O44K74	0.34 g·L^-1^·h^-1^	32	Anaerobic	56
*Proteus mirabilis*	0.72 g L^-1 ^g^-1^	40	poly(vinyl alcohol) cryogels	59
*E. coli *LMG194 pBADcaiC	1,7 g h^-1 ^g^-1^	42	Overexpression of CaiC	26
*E. coli *BW25113 *ΔaceA*	2.88 g h^-1 ^g^-1^	24	Suppression of glyoxylate shunt	26
*E. coli *BW25113 *ΔaceK*	3.17 g h^-1 ^g^-1^	22	Suppression of glyoxylate shunt	26

**Resting Cells**				

**Strain**	**Productivity**	**Molar yield (%)**	**Comment**	**Ref.**

*E. coli *pT7-5KE32	4.3 g·L^-1^·h^-1^	53.2	Anaerobiosis	61
*E. coli *pT7-5KE32	11.3 g·L^-1^·h^-1^	70.1	Aerobiosis	61
*Proteus sp.*	6.1 g·L^-1^·h^-^	50	Aerobiosis	79
*Agrobacterium *sp. 525a	1,2 g·L^-1^·h^-1^	38	D-Carnitine	781
*E. coli *O44K74	0.55 g·L^-1^·h^-1^	44	D-carnitine	14
*E. coli *pT7-5KE32	0.90 g·L^-1^·h^-1^	40	D-carnitine	62
*E. coli *O44K74	2.6 g·L^-1^·h^-1^	65	Salt Stress	71
*E. coli *pT7-5KE32	3.2 g·L^-1^·h^-1^	80	Salt Stress	71
*E. coli *O44K74	3.6 g·L^-1^·h^-1^	89	Polyethylenimine Permeabilized Cells	76
*E. coli *pT7-5KE32	3.8 g·L^-1^·h^-1^	94	Polyethylenimine Permeabilized Cells	76
*Proteus sp.*	1.6 g·L^-1^·g^-1^·h^-1^	68	Triton X-100 Permeabilized Cells	79

**Continuous Processes**				

**Strain**	**Productivity**	**Molar yield (%)**	**Comment**	**Ref.**

*Escherichia coli *O44K74	0.3 g·L^-1^·h^-1^	---	Immobilized in polyacrylamide	83
*Escherichia coli *O44K74	1.8 g·L^-1^·h^-1^	26	Immobilized in glass beads or polyurethane foam	56
*E. coli *O44K74	6.2–12 g·L^-1^·h^-1^	40	Cell recycle	55,63
*E. coli *pT7-5KE32	1.2 g·L^-1^·h^-1^	24	Cell recycle (plasmid loss after 4–6 days)	63
*E. coli *pT7-5KE32	0.71 g·L^-1^·h^-1^	10	Immobilized in kappa-carrageenan	63
*Proteus sp.*	40.5 g·L^-1^·h^-1^	35–50%	Cell-recycle	58
Proteobacteria	5.4 g·L^-1^·h^-1^	90–95	Cell-recycle	84

In order to optimise the continuous production of L-carnitine, the suitability of using a cross-flow filtration module (with flat-sheets) and hollow-fiber devices for the retention of cells within the system has also been checked. To attain high productivities by means of high cell density continuous cultivation, microfiltration reactors seem to be suitable since the accumulation of toxic by-products is avoided [54,55]. Further, membrane reactors can also be used with alternating growing and resting cells cycles so that a cheaper process might result [55,58,63].

Thus, systems equipped with three different membrane types were studied: cellulose membranes of 300 kDa nominal cut-off, polysulphonated polysulphone membranes of 0.2 μm nominal cut-off and ceramic membranes of 0.1 μm nominal cut-off. The continuous reactor system was a CSTR plugged into the membrane system, in this way *in situ *separating cells and the product. Operation was performed at a 1.5–1.8 h^-1 ^dilution rate, using the *E. coli *O44K74 strain (Table 3). No important differences were observed and it was concluded that membrane composition had little influence on reactor performance as similar productivities were obtained.

In order to compare operational stability for different membrane configurations, a new reactor set-up based on a hollow-fiber cartridge module with fibers made of polysulphone, with a total surface available of 0.03 m^2^, was used. Throughout the set of experiments, high-cell density membrane reactors were run at different dilution rates and growing and resting periods were alternated. In this way, the continuous production of L-carnitine was as high as 12 g·L^-1^·h^-1 ^and the operation remained stable for long periods of time [63].

Moreover, these membrane reactors have also been shown to be useful for high cell density cultivation of *Proteus sp., *reaching up to 35–45 g·L^-1 ^dry biomass retained, similar to values reported for other microorganisms [54,58]. In addition, the suitability of this bioreaction system for biotransformation was demonstrated by reaching 35–50% yield of conversion and productivities as high as 40.5 g·L^-1^·h^-1 ^[58]. However, the susceptibility of *Proteus sp. *to this bioprocess scheme was shown to be high, as observed by the high biomass decay during resting cycles, similar to what has been reported in batch systems [59] and, thus, optimization work should be performed in order to stabilize cells.

With both microorganisms, performance was much better in terms of both biomass and process stability when hollow fiber rather than flat sheet membranes were used, the former permitting long-term cultivation experiments. Furthermore, the degree of adherence of the cake layers to the membranes was lower in the hollow fiber system. These features led to increased productivity with *Proteus sp.*, while with *E. coli *lower productivities were attained, despite the higher biomass levels, due to a lower degree of induction of the biotransformation pathway [63]. Thus, the influence of membrane composition should be checked in each particular case, since interspecies variations might occur.

Finally, it should be mentioned that, in terms of conversion, the best results so far obtained are those reported using the *Rhizobium*-like, HK1349 strain and γ-butyrobetaine as substrate. In fact, conversion in the range of 90–95% and productivities of up to 5.4 g·L^-1^·h^-1 ^have been reported in cell recycle reactors. Moreover, the process was scalable to up to 2500 L, but the production costs were high compared with the fed-batch operated process, despite the lower productivity attained (about 75% lower, 99% conversion) [84].

#### 6. Analysis of cell state in bioreactors: cellular metabolism and physiology

The conditions of bioreactor operation have a profound impact on cell physiology. For biotechnological applications, high cell densities are desirable, but metabolic limitations are more likely to arise under these conditions, even in continuously fed systems (fed-batch, semi-continuous and continuous reactors), putting cell viability at risk. Under these conditions, cells can enter doubtfully viable, non-dividing states [85] which, nevertheless, can be metabolically active [57]. The application of flow cytometry allows the cell physiological state (as determined by differential uptake of fluorescent probes by cells) to be related with bioreactor performance, thus linking the micro- and macro-scale levels of the biotransformation.

**Figure 4:**
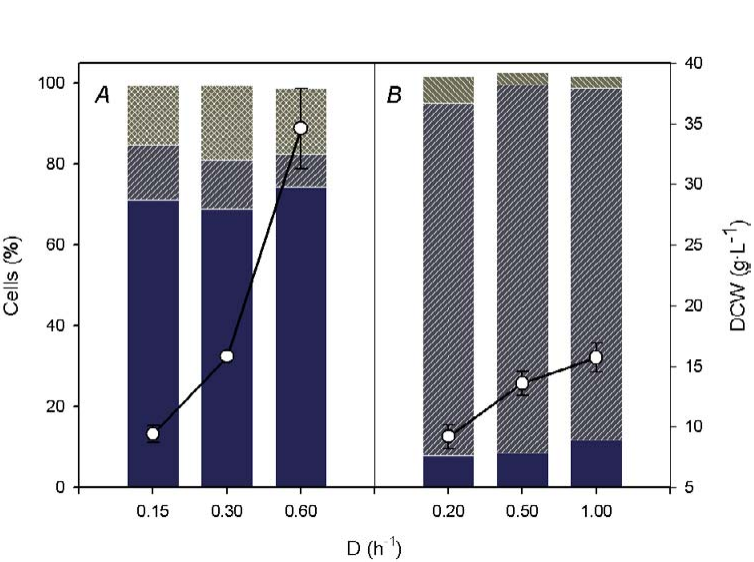
Physiological state of *E. coli *strains during continuous L-carnitine production: A) High density cell-recycle reactor using *E. coli *O44K74; B) Continuous stirred tank reactor with κ-carrageenan gel immobilized *E. coli *K38 pT7-5KE32 cells. Bars represent the amount of viable (low bar), depolarized (middle bar) and dead cells (top bar) [57,82] whereas lines represent dry cell weight.

Given the importance of the cell state in the determination of final bioprocess yield, cells have been classified into viable intact, viable but non-culturable and dead cells [86]. Thus, for optimization purposes, research on the ideal cell state for a given biotransformation needs to be carried out. Double fluorescent staining of cells with propidium iodide and bis-oxonol [86] allows us to determine population kinetics in bioreactors. In resting cell systems, cell physiology decays rapidly as a result of the lack of culture medium [82]. In addition, trimethylammonium compounds, which are osmoprotectant [70], have a protecting effect on cells during the biotransformation. Surprisingly, cell state upon high cell density cultivation in cell recycle reactors was characterized by high levels of viability (Fig. 4A, less than 15% of depolarized cells in the steady state) [82]. When cells were immobilized in κ-carrageenan gel beads, a high accumulation of damaged cells was observed (less than 10–15% of cells retaining membrane polarity in the steady state) (Fig. 4B) [57]. This result is of great importance, since it underlines the fact that cells with somewhat uncertain viability can also be active producers as a result of maintaining the carnitine metabolism still active.

### Bioprocess and metabolic modelling for L-carnitine production with *E. coli *strains

Modelling is the most rational way of optimizing bioprocesses, since it not only allows for a more accurate description of production kinetics, but also for the application of optimization approaches [87] and the design of a strain improvement program [88,89]. Quantification of growth and product formation kinetics, as well as the assessment of metabolic fluxes, are a requirement for a detailed knowledge of cellular metabolism. In addition, changes in the ratios of induction of the main metabolic pathways under different reactor configurations or growth conditions should be measured both at enzyme/protein and transcript levels [30,90,91]. Taken together, these studies would enable the determination of feasible optimisation targets and might also allow the application of genetic engineering techniques [92]. Molecular biology techniques are now available for tailor-made strain development, especially in the case of certain bacteria (such as *E. coli*, *B. subtillis*, *L. lactis*) and yeasts (*S. cerevisiae*, *P. pastoris*). Moreover, of note is the effect of single-gene knock-out mutants on microbial physiology [93,94]. Below, the work carried out on metabolic and bioprocess modelling to further our knowledge of the L-carnitine metabolism is summarized.

#### 1. Link between central and secondary metabolism during biotransformation

Metabolic engineering has been defined as the purposeful modification of intermediary metabolism using recombinant DNA technology [88,89]. The term metabolic engineering was first defined by Bailey [92], who underlined the central role of Molecular Biology as a tool in Metabolic Engineering. Nevertheless, a proper cellular metabolic state can also be chosen by confining the cell in a well defined physiological state through a purposeful design of cultivation techniques [95].

Although this field deals with the redirection of metabolic fluxes for complete pathways rather than for the improvement of biotransformations performed by a single or a few enzymatic steps, the application of its concepts is of great interest as regards the turnover of energy, ATP, and the regeneration of the cofactors which are required in biotransformations [96,97]. Thus, in bioprocesses such as the transformation of trimethylammonium compounds, in which substrate and product transport and activation are associated to energy and the performance of the bioprocess involves the mobilization of cellular cofactors, strain improvement by metabolic engineering is also a strategy of prime importance, since new cell catalysts can be purposely devised [26,72]. In fact, when optimizing bioprocesses that depend on bacterial secondary metabolism, most efforts have been addressed to the increase in expression level or amplification of gene dosage in secondary pathways. However, for high cluster copy or high levels of expression, there seem to be limitations somewhere else in the metabolism, which cause a shift to a more complex regulation, as exemplified in the metabolic engineering optimization of β-lactam production [98,99]. In this case, the insufficient supply of precursors from the primary metabolism, or the accumulation [26] or insufficient production [99] and even depletion [100] of certain cellular cofactors can be pointed to as feasible control factors. Moreover, the applicability of cofactor engineering in bioprocesses in which the unbalanced regeneration of cofactors occurs has been demonstrated, both for the alteration of the cellular redox state [96,101,102] and the acetyl-CoA/CoA ratio [96,103]. Since cell physiology must not be compromised under the production conditions, metabolic alterations should be controlled in order to enable a proper functioning of the metabolic networks involved.

**Figure 5:**
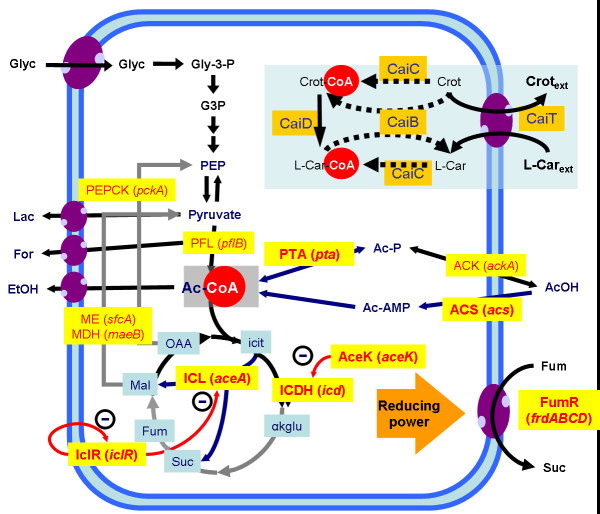
Simplified model for the interaction of L-carnitine production pathway with central metabolism of *E. coli *strains. The main pathways involved (and their codifying genes) are shown. Central metabolism: AceK (*aceK*), isocitrate dehydrogenase phosphatase/kinase; ACK (*ackA*), acetate kinase; ACS (*acs*), acetyl-CoA synthetase; ICDH (*icd*), isocitrate dehydrogenase; ICL (*aceA*), isocitrate lyase; ICLR (*iclR*), repressor of the glyoxylate shunt; MDH (*maeB*), malate dehydrogenase; ME (*sfcA*), malic enzyme; PEPCK (*pckA*), phosphoenolpyruvate carboxykinase; PFL (*pflB*), pyruvate:formate lyase; PTA (*pta*), phosphotransacetylase; FumR (*frdABCD*), fumarate reductase. L(-)-carnitine pathway: CaiB: carnitine:CoA transferase; CaiC: betaine:CoA ligase; CaiD, enoyl-CoA hydratase; CaiT, carnitine/crotonobetaine/γ-butyrobetaine transporter. Pathway regulators are shown in red. Steps which are not functional under anaerobiosis are shown with grey arrows. Adapted from [30].

In the case of carnitine production, the link or connection between central and secondary metabolisms was seen to rely on the level of cofactors [22,30]. In fact, energy depletion and an altered acetyl-CoA/CoA ratio were coupled to the end of the biotransformation. Moreover, the regulatory profiles of key central metabolic activities involved in the regulation of the acetyl-CoA/CoA ratio, such as the TCA cycle, the glyoxylate shunt and the acetate metabolism were closely interrelated and exercised a control on the biotransformation efficiency [26,30,104] (Fig. 5). Moreover, to determine control points in the metabolism of production strains, we analysed the dynamic evolution of the central metabolism after perturbations affecting the level of carbon sources, biotransformation substrate or electron acceptors. The results underlined the fact that both ATP levels and the availability of free CoA depended on cellular metabolism under production conditions [100]. Furthermore, suboptimal energetic state of *E. coli *upon high cell density cultivation conditions limited maximum productivity because of the low level of betaine activation. In addition, changes in gene expression, as reflected by altered levels of enzyme activity and external metabolite fluxes, were a result of the rapid modification of metabolite pools after pulses, revealing the ability of bacterial metabolism to redirect fluxes in order to restore the steady state conditions.

Metabolic Flux Analysis further substantiated the importance of the cellular energetic state for the biotransformation [105]. The results demonstrated a need for a high amount of ATP to be devoted to the biotransformation process. This is not only because it is consumed for the transport and activation of trimethylammonium compounds, but also because of a feasible futile cycle, which could be detected by the analysis of the network's topology. This is a consequence of the simultaneous operation of the two trimethylammonium compound carriers, CaiT and ProU [6,69] (Fig. 2A) which leads to energy dissipation. Moreover, novel strategies for the improvement of the bioprocess using functional knock-out mutants can be predicted. This is also an illustration of the fact that, in addition to the occurrence of merely metabolic control factors, such as the lack of adequate levels of cofactors or precursors for biosynthesis, transport limitations can occur during biotransformations (see previous section).

Finally, a novel application of the modelling of intracellular processes is the inclusion of signalling networks in models [106], which opens up new ways for bioprocess optimization and control strategies, widening monitoring possibilities.

#### 2. Modelling L-carnitine production: unstructured and structured reactor models

Unstructured models use simple equations with few variables, each contributing a physical meaning, while structured models use a wide number of variables which take into account that a cell is composed of many components each reacting with the others. In general, unstructured models are based on Monod's rate equations, where growth depends on biomass concentration as well as on a sole limiting substrate. Although this means a huge simplification of microbial behaviour, the ability to describe system kinetics has been widely demonstrated. An unstructured model of L-carnitine production with *E. coli *strains has been developed. In addition, the inclusion of inhibition/activation functions to describe the oxygen-dependence on substrate consumption and on the induction of L-carnitine metabolism allowed modelling of the effect of this variable on the bioprocess [64]. The model was applicable to continuous and batch bioprocesses, both with growing and resting cells, representing the link between cellular metabolic productivity and the macrokinetics of the material mass balance for the reactor. In a second step, this simple model allowed the establishment of an S-system description of the cell-bioreactor combined system, which could, in turn, be used to optimize the biotechnological setup [107]. The Indirect Optimization Method [87] was also applied, determining that the dilution rate, the initial crotonobetaine concentration, and the carnitine dehydratase activity were of critical importance for maximizing L-carnitine production. In fact, a 74% increase in the L-carnitine production rate was experimentally assessed, performance which was in close agreement with the predictions of the model. In accordance with the optimized solution, a further improvement (90% increase in the L-carnitine production rate) could be attained by increasing up to 5 times the carnitine dehydratase basal activity.

#### 3. Metabolic engineering for cell improvement: feed-back between modelling and experimental analysis of cell metabolism

Although the work described represented the first two steps in the modelling of the biotransformation process, a connection with the central and secondary metabolism was still needed to fully describe the activity of the strain. In fact, the application of optimization strategies to such unstructured models can only predict improvement strategies regarding the cultivation method. Therefore, a combined model of reactor and metabolism, linking the macrokinetics of the reactor with the cellular microkinetics, was developed, and was demonstrated to be useful for the establishment of genetic engineering strategies [108]. The model showed control points at macroscopic (reactor operation) and microscopic (molecular) levels where conversion and productivity could be increased. The optimized solution suggested to increase the levels of the CoA transferase activity, CaiB, and the protein carrier, CaiT as the main targets to improve the L-(-)-carnitine production rate, predicting an enhancement of up to three times the initial productivity [108].

**Figure 6:**
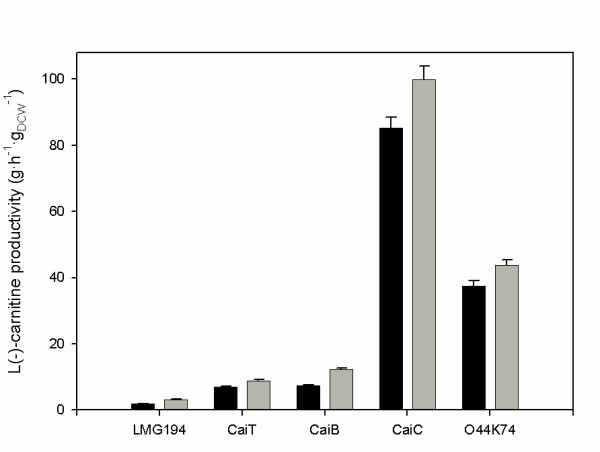
L-carnitine production with genetically engineered *E. coli *cells: effect of gene overexpression on the production of L-carnitine in *E. coli*. Experiments were performed in batch anaerobic systems in L-Broth (black bars) and L-Broth supplemented with 2 g·L^-1 ^fumarate (grey bars). Overproduction of CaiT, CaiB and CaiC was performed using pBAD24 as expression vector and *E. coli *LMG194 [F^- ^*ΔlacX74 galE galK thi rpsL ΔphoA *(PvuII) *Δara714 leu::Tn10*] as expression host. Control experiments with the non genetically engineered overexpressing *E. coli *O44K74 strain are shown for comparison. Adapted from [26,72].

**Figure 7:**
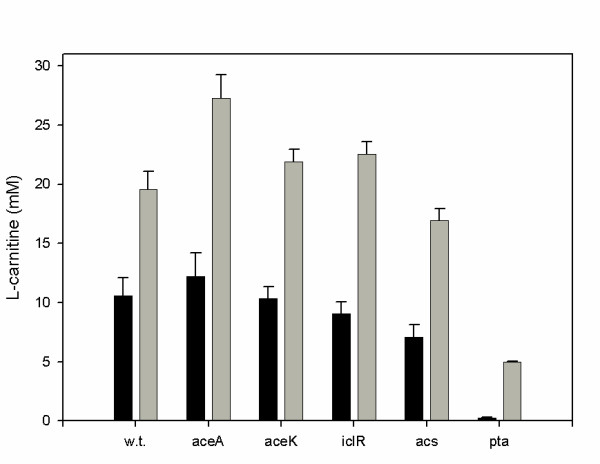
L-carnitine production with genetically engineered *E. coli *cells: effect of the deletion of *pta, acs, aceA, aceK *and *iclR *on the production of L-carnitine by *E. coli *BW25113 [*rrnB3 *Δ*lacZ4787 hsdR514*Δ(*araBAD*)*567 *Δ(*rhaBAD)568 rph-1*]. The construction of deletion mutants is described in [93]. Experiments were performed in batch anaerobic systems in L-Broth (black bars) and L-Broth supplemented with 2 g·L^-1 ^fumarate (grey bars). Adapted from [26].

These results have been experimentally validated, since by increasing the levels of CaiB and CaiT proteins in the low-producing *E. coli *LMG194 laboratory strain, L-carnitine production could be increased 3–4-fold (Fig 6) [72]. Nevertheless, the effect of CaiC overproduction, enhancing L-carnitine yield 50-fold in *E. coli *LMG194 (a 2–3-fold increase compared with the overproducing *E. coli *O44K74 strain), was not predicted by this model [26]. The main reason was that at the time that the model was built, the kinetic characteristics of this protein were not known [18]. This fact underlines an important concern: the construction of meaningful models strongly depends on the completeness and goodness of the data available. Moreover, a continuous feed-back between *in silico *and *in vivo *experimentation is necessary for the application of Metabolic Engineering and Systems Biology approaches to living systems [72]. In this context, L-carnitine production with genetically engineered *E. coli *cells with certain central metabolism encoding enzymes deleted, such as *pta *(phosphotransferase), *acs *(acetylCoA synthetase), *aceA *(isocitrate lyase), *aceK *(isocitrate dehydrogenase) and *iclR *(aceA inhibition), has been determined (Fig. 7). The results have important implications for the model built to represent this biotransformation, underlining that proper expression of the central metabolism, affecting balanced cofactor levels (acetyl-CoA/CoA in this case), influences secondary metabolism pathways [26].

### Future perspectives

The main limitation of using *E. coli *for this biotransformation process is the fact that the carnitine metabolism is induced under anaerobic conditions, since the expression of *caiF *is prevented in the presence of oxygen (Fig. 1) [4,29], and glycerol has to be used as the main carbon source, since PTS sugars inhibit its expression. In the case of *Proteus sp.*, PTS sugars also inhibit the carnitine metabolism expression, but the biotransformation occurs readily under aerobic conditions [23]. Previous work has shown that the inhibition of carnitine metabolism by oxygen can be avoided by engineering *caiF *expression [29,63,64]. Furthermore, on-going work focuses on the development of biochemical systems to avoid PTS sugars inhibition [106], carrying out the biotransformation under energetically more favourable conditions. Also, heterogenic C-sources, which are cheaper for industrial purposes, could be used, since these would not pose any diauxie problem either. This is presently under study by our group.

## Conclusion

The present knowledge on bacterial carnitine metabolism and the potential industrial application of its production methods focuses on both bioprocess development and strain optimization. This article has presented an overview on how genetically engineered and wild-type cells in their growing, resting and permeabilized states can be used for the development of biotechnological processes such as the production of L-carnitine. More importantly, the trimethylammonium compounds metabolism of *E. coli *and *Proteus sp. *was found to exercise an important degree of control on the process, which enabled the application of metabolic engineering approaches. The need for a balanced connection between the primary and secondary metabolisms, the transport of the biotransformation substrate/product and the activation of the carnitine intermediaries involved in bioprocess has been underlined. Besides this, *E. coli*'s physiology in production conditions, is now better understood and different reactor configurations for process optimization are also possible. Furthermore, the design of unstructured and structured models of the bioprocess has allowed development of novel optimization strategies, closing the iteration-cycle between the *in silico *and *in vivo *approaches of analysis of this biotransformation process.

A combination of strategies should lead to the optimized performance of the biotransformation. The choice of batch or continuous systems mainly depends on the difficulties involved in the scaling-up processes. In general, batch systems are preferred in industry, while the permeabilization, immobilization or reutilization of cells would improve bioprocess costs. The most promising guidelines for strain development would include the overproduction of the carnitine activation enzymes (CaiB and CaiC), together with the trimethylammonium compounds transporter (CaiT) and the down-regulation of the expression of the glyoxylate shunt. Furthermore, the use of chromosomal insertion technologies would ensure strain stability during the bioprocess.

Although biotransformation processes are designed on a case-by-case basis, the experimental and theoretical methodologies of Bioprocess and Metabolic Engineering are applicable to the development of any bioprocess involving whole cells. To further understanding of the functioning of any biotransformation process, state-of-the-art modelling and optimization techniques, in combination with a study of the genome, transcriptome and proteome, as well as of the regulome, will lead to new approaches to understand these bioprocesses. These approaches are currently being applied in our laboratory for the improvement of bioprocesses carried out by *E. coli *strains.

## Competing interests

The author(s) declare that they have no competing interests.

## Authors' contributions

VB and MC wrote the paper. AS, and JLI revised the manuscript critically. All authors were involved in the conception and design of experimental set up and in the analysis and interpretation of results. All authors read and approved the final manuscript.
